# Supplementary material for: Can lung ultrasound score accurately predict the need for surfactant replacement in preterm neonates? A systematic review and meta-analysis protocol
Source: PLoS One. 2021 Jul 28;16(7):e0255332. doi: 10.1371/journal.pone.0255332 (PMC8318286; doi:10.1371/journal.pone.0255332)
Supplement: Attachment — Submitted filename: final answer to PLOS lus protocol (1) (1).docx [file pone.0255332.s002.docx]

To: Dr Kazumichi Fujioka

Academic Editor
PLOS ONE	Naples 10/07/2021

Thank you for the interest in our protocol.

We now provide a revised version of the manuscript following your observations and the reviewers’ suggestions. Below you will find a point by point answer to the comments.

**EDITOR’S COMMENTS**

**1.** Please ensure that your manuscript meets PLOS ONE's style requirements, including those for file naming. The PLOS ONE style templates can be found at…….

**Answer:** the renewed manuscript strictly adheres to the journal’s style requirements

**2.** Please confirm that you have included all items recommended in the PRISMA-P checklist including:
- the full electronic search strategy used to identify studies with all search terms and limits for at least one database.
- an explanation for why the search inclusion dates begin in 2011
- an explanation of how risk of bias will be assessed

**Answer:** we closely follow all the PRISMA-P checklist.

Moreover:

-A search strategy has been now detailed from lines 88 to 96.

-The original approach to neonatal lung ultrasound was purely descriptive and no score strategy was contemplated. We published an image classification system in 2012, subsequently, Brat and coworkers adopted the numerical score that is currently widely used. We believe that a 10 years interval is appropriate to give the reader complete information and we have extended our search to June 2021 (line 86).

-Risk of bias assessment is described in the section “Risk of bias in included studies”. Changes have been made to the section to make it clearer for the reader (line 123-128).

-We added the PROSPERO registration number (lines 25 and 56) in the Abstract and Introduction sections.

 3.………..We note that you have stated that you will provide repository information for your data at acceptance. Should your manuscript be accepted for publication, we will hold it until you provide the relevant accession numbers or DOIs necessary to access your data. If you wish to make changes to your Data Availability statement, please describe these changes in your cover letter and we will update your Data Availability statement to reflect the information you provide”

**Answer:** we would change the Data Availability statement in: **Data available on request**.

4. Please include captions for your Supporting Information files at the end of your manuscript, and update any in-text citations to match accordingly. Please see our Supporting Information guidelines for more information: <http://journals.plos.org/plosone/s/supporting-information>.

**Answer:** we added caption at the end of manuscript (line 160) and updated citation in text (line 58-59)

**REVIEWERS' COMMENTS**

**Reviewer #1**

This manuscript reports a systematic review and meta-analysis concerns the accuracy of LUS, assessed on the first day of life, to predict surfactant replacement. This is a good paper that increases the existing scientific knowledge in this compound.

**Reviewer #2**

Thank you for giving me the opportunity to review this Study Protocol by Capasso L. et al. titled « Can lung ultrasound score accurately predict the need for surfactant replacement in preterm neonates? A systematic review and meta-analysis protocol."».
Overall the protocol is useful and accurate. The objectives are clearly stated, the search strategy and the statistical methodology are very well detailed, making it possible to decide on the level of proof about the studied technique with maximum bias avoidance.

I have a few comments/suggestions:

**1.** The authors aim to study the accuracy of a referenced ultrasound score, LUS (Brat et al., JAMA Pediatr 2015), to predict surfactant treatment in premature infants. Is there not a way to include other studies done with comparable objective and technique, but assessing parenchymal disease by the means of other scores ?

**Answer**: Brat et al applied to neonates with RDS a numerical score from the adult literature (Brat et al., JAMA Pediatr 2015) that has been validated versus a number of physiological variables.

This physiological correlation represents a point of strength of this non invasive tool and probably stands behind its relative popularity.

This is less true for other scores where either a formal validation is lacking or a qualitative (i.e. type 1, type 2, type 3) rather than a semiquantitative strategy was adopted .

A direct comparison, therefore, appears inappropriate.

**2**. The need for invasive mechanical ventilation, the occurrence of pneumothorax and air leaks, of bronchopulmonary dysplasia and death could be studied as secondary endpoints. These elements would provide some benchmarks for future randomized controlled trials comparing ultrasounds-guided surfactant replacement to conventional modalities, as oxygenation index or fraction of inspired oxygen.

**Answer**: the reviewer has rightly underlined relevant clinical information that will now be acquired ( cfr line 112-117).

**3**. Statistical analysis should mention term subgroup analyzes.

**Answer**: Subgroup analysis was considered as a method to be employed especially in case high heterogeneity is detected among the eligible studies, but also in the case of populations with different characteristics. The section has also been slightly rephrased to evidence this aspect and make it clearer to the reader (lines 133 – 135).

**4.** The authors should please comment on the contributions of their study protocol compared to the article "Neonatal lung ultrasonography to evaluate need for surfactant or mechanical ventilation: a systematic review and meta-analysis.", published in Arch Dis Child Fetal Neonatal Ed. 2020 Mar;105(2):164-171. doi: 10.1136/archdischild-2019-316832.

**Answer**: the mentioned manuscript has the invaluable credit to be the first review and metanalysis on the topic. The authors included the three single center studies available in 2018 where a wide interval of gestational age (term and preterm) and different causes of respiratory distress (e.g. RDS and TTN) were included.

Since then, a few pertinent manuscripts have been published, more investigation groups have been involved and the number of babies has substantially increased.

The aim of our protocol differs from Razak et al in that it is focused only on preterm neonates with RDS that need surfactant replacement (line 51-52).
